# The ambivalent nature of the relationship between lymphatics and cancer

**DOI:** 10.3389/fcell.2022.931335

**Published:** 2022-09-07

**Authors:** Joshua Choi, Ellie Choi, Dongwon Choi

**Affiliations:** Department of Surgery, Norris Comprehensive Cancer Center, Keck School of Medicine, University of Southern California, Los Angeles, CA, United States

**Keywords:** lymphatics, cancer, lymphangiogenesis, metastasis, drug delivery, immune surveillance

## Abstract

Do lymphatic vessels support cancer cells? Or are they vessels that help suppress cancer development? It is known that the lymphatic system is a vehicle for tumor metastasis and that the lymphangiogenic regulator VEGF-C supports the tumor. One such role of VEGF-C is the suppression of the immune response to cancer. The lymphatic system has also been correlated with an increase in interstitial fluid pressure of the tumor microenvironment. On the other hand, lymphatic vessels facilitate immune surveillance to mount an immune response against tumors with the support of VEGF-C. Furthermore, the activation of lymphatic fluid drainage may prove to filter and decrease tumor interstitial fluid pressure. In this review, we provide an overview of the dynamic between lymphatics, cancer, and tumor fluid pressure to suggest that lymphatic vessels may be used as an antitumor therapy due to their capabilities of immune surveillance and fluid pressure drainage. The application of this potential may help to prevent tumor proliferation or increase the efficacy of drugs that target cancer.

## Introduction

From alterations in lymphatic vessel density to increased hypoxia and interstitial fluid pressure, the distinct tumor microenvironment controls most aspects of tumor proliferation, metastasis, and anti-tumor resistance. Therefore, it has become increasingly evident that understanding the tumor microenvironment and altering it is crucial for enhancing survival rates in cancer patients.

The lymphatic system and its relationship to tumors has been a topic of heavy contention. Some argue that lymphangiogenesis correlates with increased metastasis to lymph nodes first and then to distal regions of the body (Jackson et al., 2001; Skobe et al., 2001; Padera et al., 2002; He et al., 2004; Saharinen et al., 2004; Sleeman and Thiele, 2009; Alitalo and Detmar, 2012; Swartz, 2014; Farnsworth et al., 2018). It is further debated whether lymph node metastasis is simply an indication of a more aggressive tumor or a concrete step in distal metastasis. However, other evidence suggests that lymphangiogenesis may not be associated with lymph node metastasis (Jeong et al., 2015; Asaoka et al., 2020). Finally, recent discoveries contend that lymphangiogenesis along with immunotherapy treatments may increase patient survival against tumors through immune surveillance and activation (Song et al., 2020; Sasso et al., 2021). Therefore, there is still much to be discovered about this relationship and its clinical significance.

Tumor interstitial fluid pressure (TIFP) is a topic of growing interest in the understanding of cancer and developing potential avenues of therapy. TIFP has been shown to indicate poor prognosis and increased drug resistance in patients and studies have attempted to elucidate the mechanism by which TIFP may cause problems in therapy (Milosevic et al., 2001; Heldin et al., 2004; Padera et al., 2004; Taghian et al., 2005; Rofstad et al., 2014; Schaaf et al., 2018; Böckelmann and Schumacher, 2019). Of particular interest for this review is the interplay between TIFP, hypoxia, lymphatics, and cancer metastasis. Does the increase in hypoxia affect the TIFP? Do lymphatics and lymphangiogenesis cause changes in the TIFP? Finally, does TIFP alter the rate of cancer metastasis?

In this review, we discuss the various viewpoints regarding lymphangiogenesis, cancer metastasis, and tumor interstitial fluid pressure (Table 1). We will also examine the growing view of the relationship between lymphatics and immunology specifically in the context of tumor biology. Finally, we will offer our postulations and suggest future directions of research into these topics of interest.

**TABLE 1:** The different negative and positive views of lymphatics that contribute to the relationship between cancer and lymphatics.

Negative View of Lymphatics	Positive View of Lymphatics
1. Facilitate cancer cell metastasis	1. Increase immune surveillance of tumor antigens
2. VEGF-C signaling suppresses T cells	2. Enhanced recruitment of T cells to fight tumor cells
3. VEGF increases recruitment of immunosuppressive leucocytes	3. Delay tumor growth with checkpoint inhibitors
4. Lymphatic abnormalities and recruitment of inflammatory cells contribute to fluid buildup in tumors	4. Lymphatic drainage may decrease fluid buildup in tumors

### Section 1: Current view of cancer lymphangiogenesis, chemotaxis, and metastasis

Studies have shown a strong link between lymphangiogenesis and cancer metastasis. The increase in lymphangiogenesis is correlated with an increase in the metastasis of cancer cells to the lymph nodes (Jackson et al., 2001; Skobe et al., 2001; Padera et al., 2002; He et al., 2004; Saharinen et al., 2004; Sleeman and Thiele, 2009; Alitalo and Detmar, 2012; Swartz, 2014; Farnsworth et al., 2018). It was unknown whether this metastasis is simply an indication of tumor aggressiveness or a concrete step in the cancer’s invasion of other vital organs, but recent evidence has suggests that the latter is true (Brown et al., 2018). In this section, we highlight the molecular mechanisms of lymphangiogenesis and its effect on cancer metastasis and immune functioning.

VEGF-C and VEGF-D are pro-polypeptides that undergo proteolytic cleavage to influence early lymphatic vessel development and proliferation through its interaction with receptors VEGFR-3 and VEGFR-2 (Joukov et al., 1997; Stacker et al., 1999). Although it is expressed in endothelial cells (ECs) of blood vessels during development, VEGFR-3 is later expressed solely in lymphatic endothelial cells (LECs), which makes it useful as a lymphatic marker. Through their ligands (VEGF-C and VEGF-D) VEGFR-2 and VEGFR-3 facilitate lymphangiogenesis (Jeltsch et al., 1997; Veikkola et al., 2001). One study shows that while VEGFR-3 causes the sprouting of lymphatic vessels, VEGFR-2 modifies the process by inducing the enlargement of the vessels (Wirzenius et al., 2007)**.** However, many other studies have shown that VEGFR-2 is also capable of lymphangiogenesis, and that VEGF-A overexpressing tumors induced lymphangiogenesis through VEGFR-2, including sentinel lymph node lymphangiogenesis (Hirakawa et al., 2005; Dellinger et al., 2013). The overexpression of a VEGFR-3 antagonist was shown to cause apoptosis in LECs and regression of intact lymphatic vessels in mouse embryonic skin, suggesting that VEGFR-3 is necessary for the proper development and maintenance of lymphatic vessels and LECs (He et al., 2002; Karkkainen et al., 2004).

VEGFR-3 is important in lymphangiogenesis, and cancer cells utilize this mechanism of VEGF-C to promote metastasis (Jackson et al., 2001; He et al., 2004; Saharinen et al., 2004; Alitalo and Detmar, 2012). A study showed that experimentally induced overexpression of VEGF-C in breast cancer cell lines, MDA-MB-435 and MCF-7, caused increased tumor spread to regional lymph nodes (Skobe et al., 2001; Mattila et al., 2002). Furthermore, the use of VEGFR-3-Ig, a VEGFR-3 antagonist, in a mammary tumor model in rats inhibited lymph node metastasis (He et al., 2002). This experiment did not affect lymphatic vessels that were already present, suggesting that cancer cells induce new lymphatic vessel development to facilitate metastasis into the lymph nodes (He et al., 2002; He et al., 2004).

Currently, cancer metastasis through lymphatics is outlined in the sequential model whereby the primary tumor induces lymphangiogenesis and lymphatic remodeling (Farnsworth et al., 2018). Peritumoral lymphatic vessels specifically can collect disseminating tumor cells and contribute to metastasis while intratumoral lymphatic vessels have been shown to be dysfunctional (Gasparini et al., 1994; Ji, 2006). After entering the peritumoral lymphatic vessels, disseminating cancer cells access regional then distal lymph nodes, eventually entering the thoracic duct to access other organs *via* blood vessels (Brown et al., 2018; Farnsworth et al., 2018). Lymphangiogenesis also occurs in distal sites such as the lungs to promote further metastasis (Ma et al., 2018). At any point in this process, the cancer cells may enter the bloodstream to follow the hematogenous model, which outlines the spread of cancer cells through blood vessel invasion (Farnsworth et al., 2018). In fact, the metastatic cells in the lymph node can enter the surrounding blood vessels which cause distant metastases (Brown et al., 2018; Pereira et al., 2018). However, the sequential model of cancer metastasis is under debate due to clinical evidence that suggests that not all patients benefit from the removal of regional lymph nodes (Farnsworth et al., 2018). There have been many studies in favor and opposition to the idea that lymphatic metastasis contributes to distant metastasis. In both mouse models and clinical studies, it has been shown that lymphatic metastasis was correlated with distant metastasis (Leemans et al., 1993; Brown et al., 2018; Ma et al., 2018; Pereira et al., 2018). Other studies have shown that this correlation may not be the causation, suggesting that distant metastases are mainly seeded by the primary tumor directly rather than through the lymph node (Naxerova et al., 2017; Tang et al., 2021). More studies should be done to elucidate a solution to this apparent paradox. Another subject of debate has been the mechanism of cancer invasion into the lymphatic vessels. One explanation suggests that the increase in lymphatic surface area due to lymphangiogenesis increases contact between lymphatics and cancer (Saharinen et al., 2004). Another suggests that the high tumor interstitial fluid pressure supports tumor cell entry into lymphatic vessels (Saharinen et al., 2004; Ji, 2006).

One hypothesis that is gaining increasing support is the idea that tumor cells are able to utilize chemokine signaling to infiltrate lymphatics (Farnsworth et al., 2018). One of the primary functions of the lymphatic system is immune cell trafficking. As such, the factors that contribute to lymphatic development are involved in chemokine signaling and leukocyte migration. The glycosaminoglycan hyaluronan (HA) and its receptor Lyve-1 have been found to play major roles in this trafficking (Jackson et al., 2001; Du et al., 2013; Jackson, 2019). HA undergoes constant turnover with a half-life of about 24 h, which leads to differential effects of HA before and after degradation. Before degradation, HA is expressed on leukocytes and creates a path for extravasation and migration through the cortical and medullary sinuses (Jackson et al., 2001). HA is degraded in lymph nodes and these smaller fragments of HA are involved in angiogenesis and chemokine release. LYVE-1 is an HA receptor in the Link superfamily which resembles CD44 cell-surface receptors (Jackson et al., 2001). Although it is expressed minimally in liver, spleen, and lung ECs, LYVE-1 is predominantly found in LECs and is therefore used as a lymphatic vessel marker. The interaction of HA and LYVE-1 allows for the trafficking of dendritic cells (DCs). This is supported by evidence that shows that when Lyve-1 is knocked out, the migration of DCs is impaired (Jackson, 2019). In addition to DCs, HA expression has been found in both macrophages and T cells, indicating the widespread role of the Lyve-1:HA axis in the trafficking of a variety of immune cells (Du et al., 2013; Jackson, 2019).

Cancer cells manipulate the chemotactic interaction between Lyve-1 and HA to migrate towards lymphatic vessels (Du et al., 2013). Lyve-1 and the expression of HA on tumor cells have been highly correlated with lymph node metastases. In one study, it was shown that breast cancer cells with high expression of HA formed cable structures with Lyve-1 (Du et al., 2013). This result suggests that the presence of HA on the surface of cancer cells may enhance metastasis by increasing adherence to lymphatic vessels through its interaction with Lyve-1 (Du et al., 2013). In addition to this mechanism, tumor cells respond to the chemokine CCL21, which facilitates chemotaxis in CCR7+ expressing cells. By expressing CCR7+, tumor cells migrate toward the lymphatic endothelium thereby promoting lymph node metastasis (He et al., 2004; Sleeman and Thiele, 2009; Farnsworth et al., 2018). Furthermore, CXCR4 signaling is utilized in melanoma cell metastasis through lymphatic vessels. Lymphatic endothelial cells stimulate the migration of CXCR4+/CD133+ melanoma cells toward lymphatic vessels, and blocking CXCR4 signaling inhibits this metastasis (Kim et al., 2010).

Studies have shown that not only do cancer cells manipulate immune signaling for chemotaxis (He et al., 2004; Sleeman and Thiele, 2009; Farnsworth et al., 2018), they also are able to suppress the body’s immune response (Sleeman and Thiele, 2009; Farnsworth et al., 2018). Evidence has shown that tumors reduce the number of DCs, CD4^+^ T cells, and CD8^+^ T cells in sentinel lymph nodes (Sleeman and Thiele, 2009). The expression of immunosuppressive ligands and leucocytes further impair the body’s immune system and the tumor’s use of VEGF-C/VEGFR-3 signaling appears to aid in the suppression of CD8^+^ T cells and recruitment of additional immunosuppressive leucocytes (Lund et al., 2012; Fankhauser et al., 2017; Farnsworth et al., 2018). The expression of VEGF-C in the tumor microenvironment recruits CCL21 expressing cells and naïve T cells, and tumor associated lymphatic vessels express PD-L1, which leads to an immunosuppressive environment (Dieterich et al., 2017; Fankhauser et al., 2017; Farnsworth et al., 2018).

One of the most recent developments in our understanding of the formation of tumor lymphovasculature comes from evidence suggesting that myeloid-derived lymphatic endothelial progenitors promote lymphatic vessel formation and are involved in metastasis (Zumsteg et al., 2009; Ran and Wilber, 2017; Volk-Draper et al., 2019). Immature myeloid cells from the bone marrow migrate to the tumor environment in response to VEGF-A and subsequently increase lymphangiogenesis by integrating into pre-existing lymphatic vessels near the tumor and differentiating into lymphatic endothelial cells (Schoppmann et al., 2006; Zumsteg et al., 2009; Lee et al., 2010; Ding et al., 2012; Tawada et al., 2014). This increase in lymphangiogenesis then promotes metastasis of tumor cells (Zumsteg et al., 2009; Ding et al., 2012; Ran and Wilber, 2017; Volk-Draper et al., 2019).

Therefore, the prevailing view of the relationship between lymphatics and cancer is that cancer cells invade the lymph nodes, thereby facilitating the metastasis of cancer to other areas of the body. While the exact mechanism of cancer cells dissemination is not currently known, there are many explanations that attempt to elucidate the mechanism of cancer cell invasion into the lymph nodes. Of such explanations, one that we have highlighted is the cancer cells’ use of chemokine signaling to migrate towards and into the lymph nodes. Next, we will introduce a view of lymphatic oncology that opposes the prevailing view.

### Section 2: Opposing view of lymphangiogenesis and metastasis

As mentioned above, the predominant view of lymphovascular invasion is the idea that lymphangiogenesis mediates metastasis and that the use of antiangiogenic therapy is useful in clinical settings for patients with metastatic cancers (Jackson et al., 2001; Skobe et al., 2001; Padera et al., 2002; He et al., 2004; Saharinen et al., 2004; Sleeman and Thiele, 2009; Alitalo and Detmar, 2012; Farnsworth et al., 2018). However, clinical evidence has shown that these drugs are often ineffective in adjuvant settings and in preventing lymph node metastasis (Jeong et al., 2015). New studies have attempted to explain this discrepancy and challenge the current view of lymphangiogenesis-mediated metastasis.

Jeong et al. (2015) investigated the use of antiangiogenic therapy against tumor cells that have already undergone lymph node metastasis. Surprisingly, they found that levels of VEGF-C and VEGF-D, mediators of lymphangiogenesis, were lower in tumor draining lymph nodes (LNs) than tumor-negative LNs. Furthermore, they found that blood vessel density was also lower in metastatic tumors than in tumor-negative LNs, but also noted that lymphatic vascular area was greater in nonmetastatic tumor draining LNs compared to LNs of metastatic lesions (Jeong et al., 2015). These results indicate that the migration of tumor cells into the lymph node may induce a regression of lymphatic vasculature in the metastatic tumor draining LN. Notably, this study, in addition to the work of Skobe et al. (Skobe et al., 2001) indicate that lymphatic vessel density increases in or around the tumor (Jeong et al., 2015). Together, tumor cells induce lymphangiogenesis to promote metastasis to the lymph node, but later regress lymphatic vasculature in the metastatic tumor draining LNs.

Other evidence suggests that lymphangiogenesis may not be correlated with lymphovascular invasion (Asaoka et al., 2020). Asaoka et al. found that lymphangiogenic markers were not found at higher levels in tumors with lymphovascular invasion than those without (Asaoka et al., 2020). Specifically, they found that the expression of PDPN, Prox1, Lyve-1, VEGF-C, SPHK1, and S1PR1 was not increased in these cells (Asaoka et al., 2020). This suggests that lymphatic vessel invasion and lymphangiogenesis may be independent and noncorrelated processes. Furthermore, they failed to observe an increase in lymphatic vessel density in lymphovascular invasive tumor cells compared to those that did not metastasize into the lymph nodes (Asaoka et al., 2020). One limitation of this study, however, is that these were done for intratumoral vessels without acknowledging the presence of peritumoral lymphatic vessels. Furthermore, this study did not assess the presence of protein expression of lymphatic markers in the lymphatic endothelial cells and the correlations seen involving lymphatic vessel density are limited due to the uneven distribution of lymphatic vessels throughout the tumor. Therefore, more research must be done to examine whether these results can be replicated in peritumoral lymphovascular invasion, to verify the levels of protein expression for the points made by Asaoka et al., and to understand how these results relate to previous studies showing a positive correlation between lymphangiogenesis and lymph node metastasis (Jackson et al., 2001; Skobe et al., 2001; Padera et al., 2002; He et al., 2004; Saharinen et al., 2004; Sleeman and Thiele, 2009; Alitalo and Detmar, 2012; Swartz, 2014; Farnsworth et al., 2018).

In one investigation using immunohistochemistry, Zhang et al. (2015) determined that tumor invasiveness, not lymphangiogenesis was associated with increased metastasis in breast cancer patients. The degradation of extracellular matrix components is essential in tumor metastasis and the breakdown of these components is associated with tumor aggression (Zhang et al., 2015). Zhang et al. found that the level of MMP-9, which degrades collagen, was positively correlated with lymph node metastasis in younger women. At the same time, VEGF-C, which increases lymphangiogenesis, and lymphatic vessel density were not correlated with lymph node metastasis in both younger and older women (Zhang et al., 2015). These results suggest that lymph node metastasis occurs because of tumor invasiveness rather than increased access to lymphatic vessels through lymphangiogenesis in younger women (Zhang et al., 2015). Furthermore, the lack of correlation between VEGF-C, lymphatic vessel density, and metastasis indicates that there may be a separate mechanism other than these or tumor aggression that may cause lymph node metastasis in older women.

These studies suggest that lymphatic vessels regress after tumor cells have already invaded the lymph node and that lymphangiogenesis may not be associated with lymph node metastasis (Jeong et al., 2015; Zhang et al., 2015; Asaoka et al., 2020). These studies further imply that more research needs to be done to understand the relationship between lymphangiogenesis, cancer cells, and lymph node metastasis. In the next section, we will highlight some more recent studies that elucidate and elaborate on this complex relationship.

### Section 3: A paradigm shift: Lymphatic facilitation of immune surveillance

In the first section we discussed the evidence supporting the hypothesis that cancer cells use immune chemotactic signaling to migrate toward lymph nodes and that they may also use lymphangiogenic signaling molecules such as VEGF-C to induce immunosuppression in the primary tumor (He et al., 2004; Sleeman and Thiele, 2009; Farnsworth et al., 2018). However, the presence of tertiary lymphoid organs (TLO’s) have shown the prognostic benefit of infiltrating lymphocytes. Tertiary lymphoid organs are organized lymph-node like structures that have often been seen in areas of chronic inflammation, including tumor locations (Di Caro et al., 2014; Lin et al., 2019). In colorectal cancer, non-small-cell lung cancer, breast cancer, and melanomas, TLO’s have been correlated with better clinical outcomes. However, there are also cases, such as in melanomas, in which TLO’s have caused immunosuppression in the tumor microenvironment (Shields et al., 2010; Di Caro et al., 2014; Lin et al., 2019). Therefore, TLO’s should be further studied to elucidate the mechanisms that explain when TLO’s may enhance tumor rejection or facilitate immunosuppression.

Similar to the results seen in positive prognoses in TLO forming tumors, a new study suggests that an increase in lymphatics may enhance the body’s immune response to tumors (Song et al., 2020). In a study conducted by Song et al. (2020), it was proposed that the use of a lymphangiogenic factor and a checkpoint blockage may enhance immune surveillance of glioblastoma multiforme (GBM) and increase survival rates in mice. This was confirmed by using VEGF-C mRNA and anti-PD-1 antibodies on GBM mice, which not only increased the mice survival rate, but also caused a regression of the tumor (Song et al., 2020). Notably, this study utilized immunotherapy as a complement to increased VEGF-C due to the immunosuppressive effects of lymphatic vessels (Dieterich et al., 2017; Bordry et al., 2018). Thus, the positive effects of VEGF-C expression were maintained while reducing the negative effects of immunosuppression by the lymphatic vessels. Specificially, the mechanism of mouse survival was increased immune surveillance through T cell priming to cancer cells resulting in an increased rate of T cell infiltration in tumors. Furthermore, in addition to an increase in T cell priming, increased numbers of T cells and tumor cells were found in the deep cervical lymph nodes (Song et al., 2020). Therefore, the use of VEGF-C to increase lymphatic vessel sprouting and enlargement, in conjunction with immune checkpoint inhibitors may be useful in therapy due to an increase in antigen draining in the lymph nodes, thereby enhancing the immune response to cancers cells through priming T cells. However, it should be assessed whether this increase in T cell priming promotes increased survival of mice *in vivo* despite increased drainage of tumor cells into the lymph nodes-suggesting risks of metastasis (Jackson et al., 2001; Skobe et al., 2001; Padera et al., 2002; He et al., 2004; Saharinen et al., 2004; Sleeman and Thiele, 2009; Alitalo and Detmar, 2012; Swartz, 2014; Farnsworth et al., 2018).

There is further evidence to support the hypothesis that increased lymphatics may promote patient survival through an immunotherapeutic mechanism of fighting tumors. Lymphatic flow is important in creating a robust immune response to tumor cells (Farnsworth et al., 2018; Sasso et al., 2021). In fact, impaired lymphatic flow has been shown to decrease both innate and adaptive immune responses to implanted tumor cells in mice (Farnsworth et al., 2018). Therefore, the increase in lymphatic flow provided by lymphatic factors such as VEGF-C support the infiltration of tumors by lymphocytes. Additionally, it was found that the lymphangiogenesis induced by the interaction of VEGF-C and VEGFR-3 enhances the recruitment of naïve CD4^+^ and CD8^+^ T cells to primary tumors, which led to increased tumor rejection combined with immunotherapies (Fankhauser et al., 2017; Farnsworth et al., 2018). This recruitment, made possible by upregulation of CCL21, then leads to the activation of T cells in the primary tumor rather than the lymph node, leading to a strong immune response against primary tumor cells (Fankhauser et al., 2017; Farnsworth et al., 2018).

In accordance with previous studies (Farnsworth et al., 2018; Song et al., 2020), Sasso et al. (2021) injected mice with a VEGF-C vaccine created from irradiated tumor cells overexpressing VEGF-C. These tumor cells induced lymphangiogenesis in the site of injection. However, they did not increase lymphangiogenesis in the primary tumor site and died shortly after, which avoids the risk of increase cancer metastasis or tumor formation. Furthermore, the overexpression of VEGF-C was shown to increase lymphatic transport, T cell recruitment through increased levels of CCL21, and T cell activation for a variety of tumor antigens (Sasso et al., 2021). Such recruitment and activation of T cells is consistent with previous studies that show that VEGF-C increases antigen drainage and the levels of CCL21 (Farnsworth et al., 2018; Song et al., 2020). The VEGF-C vaccine has been proven to be effective both prophylactically and therapeutically. When B16 melanoma tumor cells were injected in mice after the vaccine was given, these mice had a 100% survival rate compared to the 50% survival rate of mice injected with GVAX (Sasso et al., 2021). GVAX is a vaccine that expresses the GM-CSF protein which stimulates the maturing and proliferation of various monocytes, including macrophages; it has shown promise in a variety of cancers and is already undergoing clinical trials (Nemunaitis, 2005). As such, the better results achieved using the VEGF-C vaccine shows that VEGF-C induced lymphangiogenesis is a potent activator of the host immune system to fight against subsequent melanomas. A similar result was shown when the VEGF-C vaccine was combined with anti-PD-1 antibodies, a checkpoint inhibitor (Sasso et al., 2021). Similar to the results seen in the study by Song et al. (2020). Sasso et al. (2021) found that VEGF-C in combination with a checkpoint inhibitor delays tumor growth and increases survival in mice when melanomas are injected before vaccine injection. Therefore, VEGF-C induced lymphangiogenesis may be used as a preventative measure or as treatment for tumor cells.

In the prevailing view of cancer and lymphatic cell interactions-outlined in section 1- we can see that VEGF-C was always seen as a factor supporting the growth and metastasis of cancer cells. In contrast to such belief, recent evidence shown in this section reveals that VEGF-C may aid in the suppression of cancer cells through cancer cell antigen drainage, T cell recruitment, and immune cell activation. The prospect of increasing immune surveillance by bolstering lymphangiogenesis and inhibiting tumor cell checkpoints seems hopeful and has great therapeutic potential (Song et al., 2020; Sasso et al., 2021). Lymphangiogenesis has long been associated with metastasis and poor prognosis (Jackson et al., 2001; Skobe et al., 2001; Padera et al., 2002; He et al., 2004; Saharinen et al., 2004; Sleeman and Thiele, 2009; Alitalo and Detmar, 2012; Farnsworth et al., 2018), but an avenue of immunotherapy may outweigh the risks of increased lymphangiogenesis (Farnsworth et al., 2018; Song et al., 2020; Sasso et al., 2021), especially if combined with agents that help prevent lymph node metastasis. However, the studies (Song et al., 2020; Sasso et al., 2021) that support this concept use models of glioblastoma multiforme in the brain and melanomas specifically, so more research using different tumors should be done to verify these results. Furthermore, they overexpressed VEGF-C which ultimately increased the survival rate of mice by priming T cells (Song et al., 2020; Sasso et al., 2021). The overexpression of VEGF-C in the primary tumor for other cancers, however, may not prove as effective as in the GBM microenvironment or a site distant from the primary tumor. Since this is the first study done using this approach, it is unknown whether using lymphatic vessels as a method of T cell priming will be effective for other types of cancers. Nevertheless, the relationship between lymphangiogenesis and immunotherapy should be studied further to elucidate the mechanism of lymphangiogenic enhancement of immune surveillance and its generalizability to a variety of tumors throughout the body.

### Section 4: Tumor interstitial fluid pressure opposes drug delivery and may be influenced and modified by lymphatic vessels

The topic of tumor interstitial fluid pressure (TIFP) is one of growing importance in the field of cancer treatment and tumor lymphatics. Specifically, the increase in interstitial fluid pressure caused by tumors prevents effective drug delivery to the primary tumors (Boucher et al., 1990; Chauhan et al., 2012). Many drugs are delivered to their target in the interstitial space through convection, or fluid flow, which emphasizes the importance in understanding TIFP and its role in resisting drug therapy (Heldin et al., 2004).

Different forces govern the uptake of drugs by tissues in general including cancer cells. These include Starling’s forces, or hydrostatic pressure and colloid osmotic pressure (Heldin et al., 2004). In normal tissue, the hydrostatic pressure of the capillaries, interstitial fluid osmotic pressure, and the negative interstitial hydrostatic pressure contribute to a net flow of fluid out of the capillaries. The interstitial fluid pressure (IFP) is normally regulated by controlling the tension of the extracellular matrix (ECM) through the interactions between ECM proteins of the interstitium (Heldin et al., 2004). Fibroblasts exert control over the collagen network and its binding to integrin in order to regulate the tension of the ECM. This collagen network then reduces the swelling pressure caused by HA and proteoglycans. As a result, fibroblasts are able to control the degree of IFP in a given area of the interstitial space (Heldin et al., 2004).

The ECM of cancer cells are different from that of regular tissue, which largely contributes to the high TIFP seen in the primary tumor (Heldin et al., 2004). In tumor cells, there is an increase in IFP, which includes both interstitial fluid osmotic pressure and interstitial fluid hydrostatic pressure. The increase in interstitial fluid hydrostatic pressure especially contributes to a net flow of fluid outward from the tumor, which impairs drug delivery. The mechanism of this TIFP increase is multifold. Not only do tumor cells use the fibroblast mechanism to increase TIFP, the leakiness of vessels and increase in inflammatory cells also further increase the TIFP. Blood vessels in tumor cells are very compressed and leaky, leading to reduced flow and increased fluid buildup in the tumor microenvironment (Heldin et al., 2004). It is thought that VEGF is responsible for vascular permeability and, thus, the leakiness of these blood vessels (Senger et al., 1983; Heldin et al., 2004). Furthermore, it has been suggested that lymphatic vessels are usually not present or nonfunctional inside the tumor which may exacerbate the lack of fluid drainage (Padera et al., 2002; Heldin et al., 2004). Other studies have indicated that lymphatic vessel density is associated with tumor cell invasion, suggesting that lymphatic vessels are functional in the tumor (Jackson et al., 2001; Skobe et al., 2001; He et al., 2004; Saharinen et al., 2004; Alitalo and Detmar, 2012; Farnsworth et al., 2018). However, it is not currently known whether the presence or absence of functional lymphatics creates a direct change in TIFP. The increase in overall TIFP created by cancer cells resists drug delivery by creating a flow of fluid outward that opposes fluid flow coming in and lymphatic vessels should be studied as a therapeutic mechanism to decrease fluid buildup in the tumor environment (Heldin et al., 2004).

The two main mechanisms of increased VEGF expression in tumors currently known include immune recruitment and hypoxic induction, which both ultimately increase TIFP (Senger et al., 1983; Sleeman and Thiele, 2009; Chouaib et al., 2012; Rofstad et al., 2014; Farnsworth et al., 2018). Cancer cells recruit macrophages and other immune cells which produce cytokine signals that often cause immunosuppression (Senger et al., 1983; Sleeman and Thiele, 2009; Lund et al., 2012; Fankhauser et al., 2017; Farnsworth et al., 2018). Tumor associated macrophages (TAMs) specifically are thought to express proangiogenic factors such as VEGF which allows for angiogenesis in cancer cells (Schoppmann et al., 2002; Li et al., 2016). Alternatively, abnormalities in tumoral blood vessels, such as resistance to blood flow and vascular leakiness, lead to nutrient deprivation and hypoxia within the tumor microenvironment. This hypoxia may induce expression of VEGF which increases angiogenesis and fuels the rapid proliferation of the tumor mass (Li et al., 2009; Chouaib et al., 2012; Rofstad et al., 2014). Hypoxia-inducible factor (HIF), for instance, plays a role in activating a variety of genes associated with angiogenesis, including VEGF (Li et al., 2009; Chouaib et al., 2012). The *von Hippel-Landau* (VHL) tumor suppressor gene normally targets HIF for degradation through the recruitment of E3 ubiquitin protein and destabilizes VEGF mRNA. Thus, a double allelic loss of function of VHL is associated with an increase in VEGF levels directly and indirectly through increased HIF levels (Datta et al., 2005; Baldewijns et al., 2010; Chouaib et al., 2012). As the levels of VEGF expression are increased, so are vascular leakiness, lymphatic vessel abnormalities, hypoxia, and TIFP (Senger et al., 1983; Heldin et al., 2004; Li et al., 2009; Chouaib et al., 2012).

The increase in both hypoxia and TIFP in tumors has been associated with increased metastasis as well. Currently, the increase in metastasis is attributed to metastatic migration through peritumoral lymphatic vessels created in response to hypoxia induced VEGF signaling (Rofstad et al., 2014). However, increased vessel density is only seen in the peripheral regions of the tumor while high hypoxia is seen in the central regions of the tumor. One explanation of this inconsistency is that the outward flow created by a gradient of interstitial fluid pressure brings proangiogenic factors to the periphery of the tumor mass (Rofstad et al., 2014). But this fails to explain the lack of vessel density increase in the center of the tumor. Therefore, more studies should be done to increase our understanding of the relationship between TIFP and lymphatic vessels.

Currently, there are a few potential factors to decrease the TIFP, which include VEGF inhibitors and hyaluronidase (Lee et al., 2000; Wildiers et al., 2003; Heldin et al., 2004; Tong et al., 2004). VEGF inhibitors improves the problems in vascular abnormalities and pressure and has even been shown to enhance the uptake of chemotherapy (Lee et al., 2000; Wildiers et al., 2003; Heldin et al., 2004; Tong et al., 2004). Hyaluronidase is an enzyme that degrades HA and acts quickly (within 1 h of injection) to lower the TIFP. Hyaluronidase is speculated to act by changing the environment of the ECM to relieve the interstitial fluid pressure (Brekken and de Lange Davies, 1998; Heldin et al., 2004). Since HA and VEGF may also be involved in tumor metastasis, these two inhibitors of TIFP seem to be promising tools to alleviate therapeutic resistance due to the tumor microenvironment. Paclitaxel is a chemotherapeutic agent that has also been found to decrease TIFP while also alleviating the hypoxia of the tumor microenvironment (Taghian et al., 2005). Since paclitaxel did not seem to decrease the tumor volume, it may be used in conjunction with other drugs as a form of therapy (Taghian et al., 2005).

**FIGURE 1:**
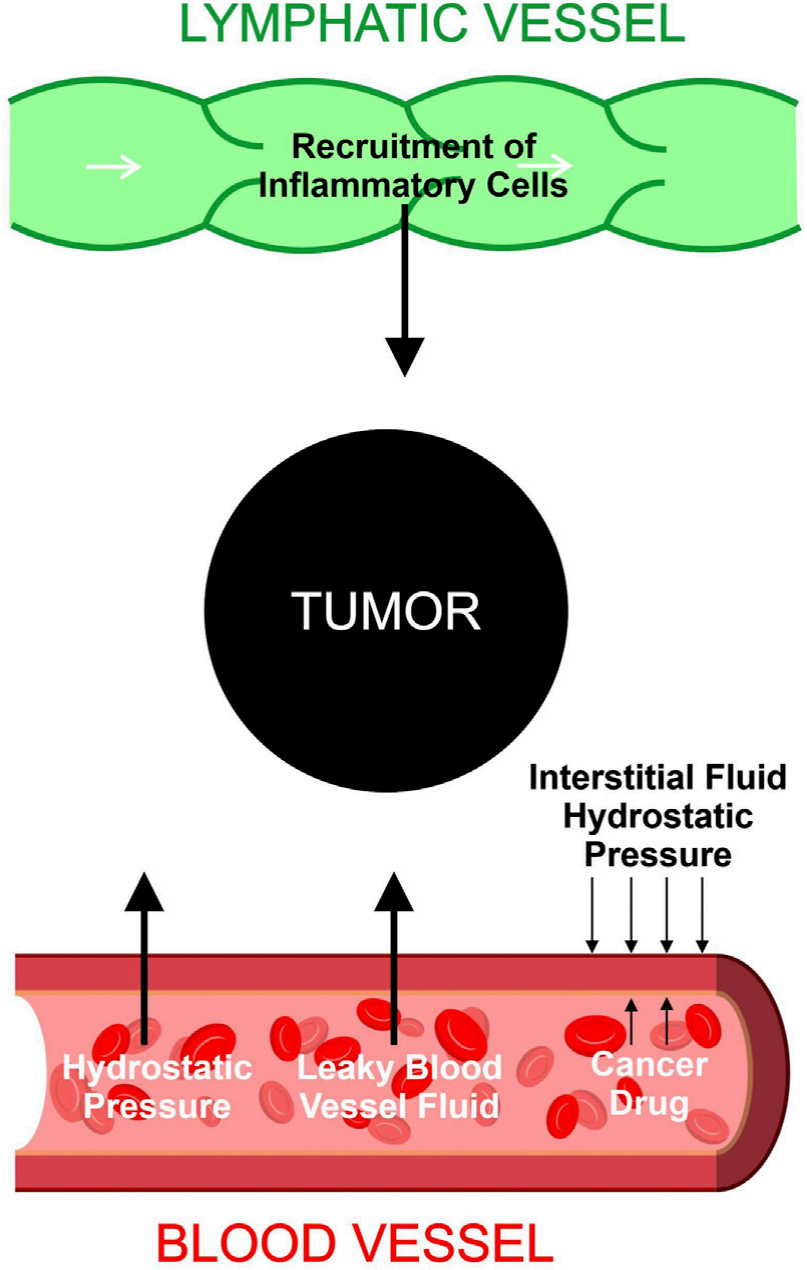
Contributions to tumor interstitial fluid buildup. Hydrostatic pressure and leaky blood vessel fluid increase the buildup of fluid inside the tumor. Lymphatic vessels recruit inflammatory cells which accumulate in the tumor microenvironment and increase the tumor interstitial fluid. The hydrostatic pressure of the interstitial fluid provides an outward force that resists drug entry into tumor cells.

**FIGURE 2:**
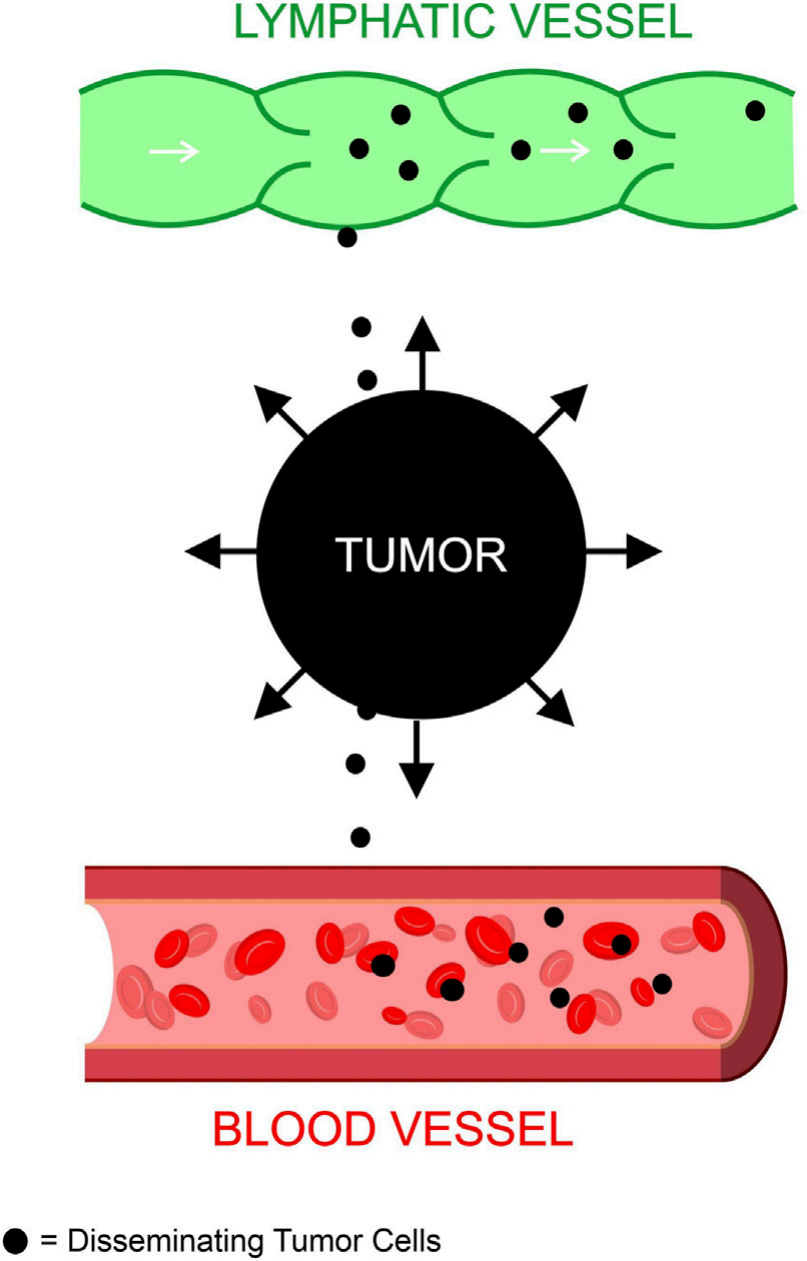
Tumor interstitial fluid pressure contributes to metastasis. The fluid buildup in the tumor microenvironment creates an outward force that disseminates tumor cells to blood and lymphatic vessels. These tumor cells enter the blood and lymph to metastasize to other areas of the body.

Based on existing studies, we formed the following postulation regarding the relationship between metastasis and TIFP outlined in Figure 1 and Figure 2. The activation of fibroblasts and the recruitment of inflammatory cells increases the TIFP. The hypoxia in the tumor microenvironment induced by the compression and dysfunction of blood vessels increases the expression of VEGF. The resulting increase in VEGF increases vascular permeability leading to a further increase in TIFP. The gradient of fluid pressure that is created through this mechanism pushes fluid outward, bringing disseminating cancer cells to the periphery where they invade the peritumoral lymph vessels, leading to lymph node metastasis.

All in all, TIFP is a profound area of interest for cancer therapy due to its opposition of drug therapies. Although the increase in TIFP is governed by a multitude of forces, the increase in VEGF, its resulting vascular leakage, and the use of lymphatic drainage may be a promising method to reduce such TIFP. Therefore, the relationship between VEGF expression and TIFP and the role of lymphatic vessels in TIFP should be further studied as a potential avenue of cancer treatment.

## Conclusion and future direction

Currently, many studies supporting the hypothesis that increased lymphatics promotes lymph node metastasis have been done by overexpressing VEGF-C. As discussed earlier, VEGF-C increases lymphatic vessel density while VEGF increases blood vessel permeability and subsequently increases the TIFP (Heldin et al., 2004). The increase in TIFP coupled with the increased lymphatic vessel density may be a cause for increased metastasis in tumor cells independent of any lymphangiogenesis that may occur. Other evidence suggests that lymphangiogenesis may not induce lymphatic metastasis due to the lack of lymphangiogenic markers in lymphovascular invasive tumor cells (Asaoka et al., 2020). This study is also limited, however, due to their examination of intratumoral vessels without consideration for peritumoral lymphatic vessels where it is now thought that lymph node metastasis takes place. More examination into a combination of antiangiogenic and lymphangiogenic factors and their effects on metastasis may provide insight to clarify the relationship between lymphangiogenesis and metastasis.

In conclusion, we speculate that increasing lymphangiogenesis through VEGF-C while introducing VEGF inhibitors and immune checkpoint inhibitors may support immunosurveillance and lower TIFP, increasing the rate of survival in patients. The lowering of TIFP may increase the uptake of the immune checkpoint inhibitors or chemotherapy while decreasing the risk of metastasis in the primary tumor. Alternatively, using a VEGF-C vaccine in a site distant from the tumor to induce lymphangiogenesis while using VEGF inhibitors within the primary tumor may increase the immune response against the tumor while lowering TIFP as well.

To test our postulation, future studies will need to assess the relationship of lymphangiogenesis with TIFP and clarify its effect on metastasis. Further studies on intratumoral lymphatics should give insight into whether such lymphatic vessels are functional, whether they contribute to metastasis, and if they have the potential to either lower TIFP or increase immune response through lymphatic drainage. Additionally, further studies should be done to verify the effect of lymphangiogenesis on immune surveillance and its effect on survival using a variety of tumors. Elucidating our understanding of such matters may be pivotal in seeing the lymphatic system, not just as a facilitator of cancer metastasis, but as an integral component of combination therapy for a variety of tumors.
